# Challenge in pathologic diagnosis of Alport syndrome: evidence from correction of previous misdiagnosis

**DOI:** 10.1186/1750-1172-7-100

**Published:** 2012-12-21

**Authors:** Xiao-dan Yao, Xin Chen, Gao-yuan Huang, Yan-ting Yu, Shu-tian Xu, Yang-lin Hu, Qing-wen Wang, Hui-ping Chen, Cai-hong Zeng, Da-xi Ji, Wei-xin Hu, Zheng Tang, Zhi-hong Liu

**Affiliations:** 1 Research Institute of Nephrology, Jinling Hospital, Nanjing University School of Medicine, East Zhongshan Road, Nanjing, 210002, China

**Keywords:** Alport syndrome, Diagnosis, Immunohistology, Renal biopsy

## Abstract

**Background:**

Pathologic studies play an important role in evaluating patients with Alport syndrome besides genotyping. Difficulties still exist in diagnosing Alport syndrome (AS), and misdiagnosis is a not-so-rare event, even in adult patient evaluated with renal biopsy.

**Methods:**

We used nested case–control study to investigate 52 patients previously misdiagnosed and 52 patients initially diagnosed in the China Alport Syndrome Treatments and Outcomes Registry e-system.

**Results:**

We found mesangial proliferative glomerulonephritis (MsPGN, 26.9%) and focal and segmental glomerulosclerosis (FSGS, 19.2%) were the most common misdiagnosis. FSGS was the most frequent misdiagnosis in female X-linked AS (fXLAS) patients (34.8%), and MsPGN in male X-linked AS (mXLAS) patients (41.2%). Previous misdiagnosed mXLAS patients (13/17, 76.5%) and autosomal recessive AS (ARAS) patients (8/12, 66.7%) were corrected after a second renal biopsy. While misdiagnosed fXLAS patients (18/23, 78.3%) were corrected after a family member diagnosed (34.8%) or after rechecking electronic microscopy and/or collagen-IV alpha-chains immunofluresence study (COL-IF) (43.5%) during follow-up. With COL-IF as an additional criterion for AS diagnosis, we found that patients with less than 3 criteria reached have increased risk of misdiagnosis (3.29-fold for all misdiagnosed AS patients and 3.90-fold for fXLAS patients).

**Conclusion:**

We emphasize timely and careful study of electronic microscopy and COL-IF in pathologic evaluation of AS patients. With renal and/or skin COL-IF as additional criterion, 3 diagnosis criteria reached are the cutoff for diagnosing AS pathologically.

## Background

Phenotypes of Alport syndrome (AS) exhibit a wide spectrum of this systemic disorder of collagen IV molecules, from mild clinical forms living to old ages to severe forms developed into end stage renal disease early in life [1-3]. The fundamental abnormalities lie in the linear and/or the conformational structures of the sub-chains of collagen IV molecules (COL-IV-α3:α4:α5 or COL-IV-α5:α6:α5), caused by different individual genotypes involving a monogenic mutation in COL4A3, or COL4A4, or COL4A5 genes [4-6]. The disease entity of AS was first delineated clinically by A. Cecil Alport in 1927 as “a dominantly inherited hereditary nephritis” characterized by hematuria and nerve deafness, affecting both the male and the female members of the family [2]. The characteristic ophthalmologic changes found in 1950s [7,8], the widespread ultra-structural changes of glomerular basement membrane (GBM) described in 1970s [9,10], and collagen-IV α-chain immunofluorescence (COL-IF) defects discovered in 1980s [11,12] made histological diagnosis of the disease more accurate, and the modes of inheritance discernable. After 1990s, genotyping added still more accuracy to the diagnosis and family counseling of AS, and nowadays, genotyping has been accepted as the gold standard for diagnosis of Alport syndrome [13,14].

However, despite these advances in understanding AS, the histology of renal biopsy (and skin biopsy for X-linked AS) still remain important in the initial evaluation of most patients suspected with hereditary nephritis, especially when renal manifestations are present. Difficulties still exist in the pathologic diagnosis in AS patients [15] and misdiagnosis remains a common event even after comprehensive clinical and pathologic evaluation. Both pediatricians and nephrologists are confronted with difficult situations in which a misdiagnosis was made in AS patients who previously underwent a renal biopsy and were then treated for other diseases. Previous studies have not addressed the risk factors for initial misdiagnosis or the data necessary to correct the diagnosis later.

To fill this gap, we conducted a retrospective study comparing initially diagnosed and previously misdiagnosed AS patients using data collected in the China AS Treatments and Outcomes Registry (CASTOR) e-system. We identified the reasons for misclassification of pathology in the previously misdiagnosed patients, and the information used to correct the diagnosis later. Odds ratios for misdiagnosis were analyzed in AS patients of different inheritance modes, and diagnostic criteria for AS discussed including COL-IF studies.

## Methods

### Patients

This study involved 104 Chinese patients with AS whose data were collected in the CASTOR e-system (designed and maintained in the Research Institute of Nephrology in Nanjing, China). Among them, 52 cases had an initial pathological diagnosis other than AS on renal biopsy during the last fifteen years in Mainland China. As controls, 52 patients were selected whose diagnosis of AS was accurately made on their first renal biopsy between 2001 and 2010 at the Research Institute of Nephrology in Nanjing. These two groups were matched by inheritance modes. General demographic data, clinical manifestations and diagnosis related parameters in the two groups are listed in Table 1.

**Table 1:** **Demographic data, clinical manifestation, and diagnostic parameters in Alport syndrome patient groups and subgroups**^**a**^

**Variable**	**Initially misdiagnosed group**				**Initially correctly diagnosed group**			
	**Total**	**mXLAS**	**fXLAS**	**ARAS**	**Total**	**mXLAS**	**fXLAS**	**ARAS**
**Case number**	52	17	23	12	52	17	23	12
**Sex (male/female)**	22/30	-	-	5/7	23/29	-	-	6/6
**Age (years)**								
At presentation	21.3 ± 12.8	20.1 ± 12.0	24.2 ± 14.0	17.3 ± 10.9	18.9 ± 12.3	14.6 ± 12.4	18.6 ± 11.4	25.3 ± 11.9
At misdiagnosis	23.4 ± 12.5	20.2 ± 11.0	27.7 ± 13.6	19.7 ± 10.5				
At final diagnosis	27.6 ± 12.6*	24.9 ± 12.0	31.6 ± 13.5*	24.0 ± 10.2	22.0 ± 11.2	19.4 ± 10.3	21.4 ± 11.8	26.8 ± 10.5
**Clinical manifestation**								
Hematuria alone	5	0	5	0	7	0	7	0
With proteinuria (>0.4 g/day)	17	4	6	7	18	4	10	4
With hypoalbuminemia (<35 g/L)	17	6	8	3	18	9	4	5
With CKD 3+ (eGFR <60 mL/min)	12	7	4	1	9	4	2	3
**Specific diagnostic criteria**								
Positive family history (proband)	39	13	18	8	37	11	19	7
Renal/skin collagen	30*^b^	14	11*	5*	43	16	17	10
Electron microscopic changes	30**^c^	10	9**	11	45	12	22	11
High tone deafness	10*	6*	3	1*	24	13	4	7
Ocular defect	11	3	5	3	6	1	1	4
**Non-specific diagnostic clues**								
Glomerular sclerosis	24	9	9	6	19	8	6	5
Interstitial foam cell	30	11	9	10	31	13	10	8
Thickness of Bowman capsule	45	17	17	11	46	16	18	12
Immunofluorescence paucity	38	14	15	9	43	16	16	11
Progressive proteinuria	37	15	11	11	35	15	10	10
Progressive hypoalbuminemia	28	12	9	7	18	10	2	6

^a^*mXLAS*, male X-linked Alport syndrome; *fXLAS*, female X-linked Alport syndrome; *ARAS*, autosomal recessive Alport syndrome.

^b^ Chi-squared test, *p* < 0.05.

^c^ Chi-squared test, *p* < 0.01.

In the misdiagnosis group, a treatment regime had been designed for every patient and carried out or followed-up for at least six months according to the misdiagnosis of the biopsy. These patients were followed as out-patients by nephrologists in the Institute or were referred to the Institute for further evaluation. Correction of diagnosis was made for every patient utilizing the diagnostic criteria and the procedures described below.

### Diagnostic criteria of alport syndrome

Every patient in this study underwent a renal biopsy either during the initial evaluation or later re-evaluation. Diagnostic criteria for AS applied in this study included the following five specific criteria, with at least three required to establish the diagnosis. These are based on the classic criteria presented by Flinter [15].

1. Typical or atypical electronic microscopic (EM) change of GBM in renal biopsy.

2. Typical or atypical COL-IF changes observed in renal and/or skin biopsy.

3. A family member as proband whose diagnosis was based on evidence of EM and/or COL-IF in renal/skin biopsy, or on a detailed family history of an X-linked mode or of autosomal recessive inheritance (defined as consanguineous parents or both parents with hematuria).

4. Definite evidence of high-tone deafness, in both ears, with or without clinical deafness.

5. Definite evidence of a lens abnormality( anterior or posterior lenticonus) and/ or flecks in the peripheral or mid-peripheral rentina area [16].

Diagnostic clues included non-specific pathologic changes and clinical characteristics that are of diagnostic value, but not required as diagnostic criteria. These included globular/segmental glomerular sclerosis, interstitial foam cell clustering, Bowman’s capsule wall thickness, a pauci-immune pattern of deposits in routine immunoflurence, progressive proteinuria and progressive hypoalbuminemia.

### Renal and skin pathology

Percutaneous renal biopsy was performed in every patient during the initial evaluation. A second renal biopsy was performed for those referred patients whose data or samples for EM or immunofluorescence studies were not available. Handling of renal and skin biopsy samples has been described in previous reports [17]. Re-examination of biopsy slides in atypical cases is facilitated by the CASTOR e-system in China which is easily accessible to pathologists and nephrologists concerned [18].

### Correction procedures

To correct misdiagnosis, procedures used included reference to proband later diagnosed pathologically, first renal biopsy slides re-evaluation, a second renal biopsy with completed evaluation (including EM and COL-IF), skin biopsy for collagen IV a-5 chain immunoflurence study, and ruling out of coexisted morbidities (including hepatitis B virus infection, Henoch-Shönlein purpura, primary hypertension, obesity and drug-induced tubulointerstitial damages).

### Statistical analysis

SPSS 16.0 was used for the statistical analysis. Baseline characteristics of patients in each AS type were compared by using the chi-squared test for categorical variables. Data are presented as the mean ± standard deviations for continuous variables and as numbers and proportions for categorical variables. To identify the predictors of misdiagnosis risk, the number of diagnosic criteria reached was treated as a dichotomous variable (<3 vs. 3+). Logistic regression was performed to conduct univariate and multivariate analysis. All tests were two-sided. A p-value of <0.05 was considered significant.

## Results

### Patients’ general data

This cohort of misdiagnosed patients represents 13% of the 398 patients whose data are completely retrieved in the CASTOR e-system. Table 1 lists general characteristics, clinical manifestations, specific diagnostic criteria reached and non-specific diagnostic clues in the two groups. There was no significant difference in average age at presentation between the two groups or among the subgroups. A difference of significance was found in the average age at final diagnosis between the two groups and between the two female XLAS subgroups (both p < 0.05). Differences of significance were also found between the percentages of specific criteria by EM (p < 0.01) and COL-IF (p < 0.05) in the two female XLAS sub-groups. Male XLAS patients in the control group had a higher rate of detected high-tone sensorineural deafness (p < 0.05). No significant difference was found in any of the other diagnostic parameters between the two groups.

### Pathologic misclassification

In Table 2 the incorrect biopsy diagnosis is shown in subgroups of different modes of inheritance. Diffuse mesangial proliferation are predominant in both groups, while female XLAS patients were more likely to be misdiagnosed as focal segmental glomerulonephritis, with some patients found to have immunoglobulin A and/or immunoglobulin M deposits, atypical membranous changes, prominent tubulointerstial changes and ischemic vascular changes.

**Table 2:** **Misclassifications of pathology in the initially misdiagnosed group**^**a**^

**Corrected diagnosis**	**Previous misdiagnosis**	**N**	**% in subgroup**	**% in whole group**
**fXLAS**		**23**		44.2
	FSGS	8	34.8	15.4
	MsPGN	5	21.7	9.6
	IgAN (mesangial proliferation)	4	17.4	7.7
	TBMN	3	13.0	5.8
	MCD	2	8.7	3.8
	IgMN	1	4.3	1.9
**mXLAS**		**17**		32.7
	MsPGN	7	41.2	13.5
	IgAN (MsP)	2	11.8	3.8
	IgAN (with FGS)	2	11.8	3.8
	IMN	2	11.8	3.8
	FSGS	2	11.8	3.8
	HTN	1	5.9	1.9
	D_TIN	1	5.9	1.9
**ARAS**		**12**		23.1
	MCD	2	16.7	3.8
	MsPGN	2	16.7	3.8
	MN	2	16.7	3.8
	IgAN (mesangial proliferation)	1	8.3	1.9
	TBMN	1	8.3	1.9
	IgM nephropathy	1	8.3	1.9
	MPGN	1	8.3	1.9
	Pregnancy related nephropathy	1	8.3	1.9
	Obesity related nephropathy	1	8.3	1.9

^*a*^*FSGS*, focal segmental glomerulosclerosis; *MsPGN*, mesangial proliferative glomerulonephritis; *IgAN*, IgA nephropathy; *TBMN*, thin basement membrane nephropathy; *MCD*, minor change disease; *IMN*, idiopathic membranous nephropathy; *HTN*, hypertensive nephropathy; *D-TIN*, drug-induced tubulointerstitial nephritis; *MPGN*, membranous proliferative glomerulonephritis.

### Correction procedures

For each patient in the misdiagnosis group, one major finding and one or more minor findings were utilized to correct the diagnosis. Different major findings were used more often in different subgroups (Table 3). In female XLAS patients the diagnosis was frequently corrected after a family member was diagnosed or when re-examination of previous biopsy slides confirmed the presence of atypical changes in both EM and COL-IF studies. The correct diagnosis in male XLAS patients and in ARAS patients usually resulted from a second renal biopsy that included complete evaluation in both EM and COL-IF studies.

**Table 3:** Procedures for the correction of pathologic misdiagnosis in previously diagnosed group

**Subtypes**	**Primary procedures**	**N**	**Secondary procedures**	**N**
**fXLAS**	Correction after proband diagnosed	8	plus skin biopsy	3
			plus recheck of EM	5
			later found FH	6
			rule out confounding factors	2
			later developed ear/eye damage	1
	Recheck of EM/COL in renal tissue during follow-up	10	recheck EM in renal tissue	8
			recheck kidney collagen stain	2
	Renal re-biopsy with EM/COL exams	3	rule out confounding factors	2
			plus skin biopsy	1
	Skin biopsy alone	2		
**mXLAS**	Correction after proband diagnosed	1	plus skin biopsy	1
	Renal re-biopsy with EM/COL	13	plus skin biopsy	12
			later found FH	5
	Skin biopsy alone	3	later developed ear/eye damage	2
			plus recheck of EM/COL	3
**ARAS**	Renal re-biopsy with EM/COL exams	8	later found FH	6
			rule out confounding factors	4
	Recheck EM/COL exams in renal tissue	3	later found FH	1
	Proband	1	later found FH	1

N, patients number; *mXLAS*, male patients with X-linked Alport syndrome; *fXLAS*, female patients with X-linked Alport syndrome; *ARAS*, autosomal recessive Alport syndrome FH, positive family history; EM, electronic microscopy; COL,collagen-IV immunofluorescence.

### Risks for misdiagnosis

In order to identify factors which increased the risk of misdiagnosis, logistic regression was used (Table 4). The presence of three or more diagnostic criteria was used as the reference. The presence of less than 3 diagnostic criteria increased the risk of misdiagnosis 3.29-fold in all patients and 3.90 fold in female XLAS patients respectively. Even when a pathologically diagnosed proband was present in the family, the risk of misdiagnosis when less than 3 pathologic criteria were present still remained 3.82 for female XLAS patients and 3.27 for the whole group. The presence of at least three diagnostic criteria can therefore be used as a standard for diagnosis of AS in these patients evaluated with renal biopsy.

**Table 4:** **Predicting misdiagnosis risk by number of diagnostic criteria reached and types**^**a**^

**AS Type**	**N**	**Diagnostic criteria reached**		**< 3 vs. 3+**			
		**< 3 components**	**3+ components**	**OR**	**95L**	**95U**	***p*value**
Total	104	46	58	**3.29**	**1.42**	**7.60**	**0.005**
fXLAS	46	20	26	**3.90**	**0.96**	**15.89**	**0.057**
mXLAS	34	15	19	2.20	0.51	9.49	0.291
ARAS	24	11	13	2.37	0.37	15.19	0.363

^a^*AS,* Alport syndrome; *fXLAS*, female X-linked Alport syndrome; *mXLAS*, male X-linked Alport syndrome; *ARAS*, autosomal recessive Alport syndrome; *OR,* odds ratio. L for two sides analysis, and U for single side analysis.

## Discussion

The diagnosis of AS can be difficult because of the wide spectrum of both clinical and pathologic phenotypes that are seen [19,20]. Diagnosis in an adult patient is less difficult than in children because progression has usually occurred, making other diagnoses less likely. However, even in adults accurate diagnosis can be difficult, because of later-onset phenotypes, non-specific renal manifestations complicated by other coexistent morbidities, prominent non-specific light microscopic findings. This was especially true when family history were not easily available or changes of EM and COL-IF were atypical or not available [20].

Table 2 shows the percentages of patients misdiagnosed with different inheritance modes and the incorrect diagnoses they received. About 11% of AS patients with renal pathology data retrieved in the CASTOR e-system were previously misdiagnosed: 27.7% in female XLAS, 17.7% in ARAS, and 6.9% in male XLAS patients. The Patients included in the present study were adolescent or adult, evaluated at an average age of about 20 years, suggesting some were in a late-onset subgroup. In the previously misdiagnosed group, there was a 4-year interval between the misdiagnosis and the correction. This study did not include patients of autosomal-dominant mode because of the small number of cases and the uncertainty of inheritance mode determination.

Renal manifestations at initial evaluation did not differ between the misdiagnosed group and the diagnosed group. Although the age at onset was not significantly different, later evaluations were associated with the previously misdiagnosed group, especially female XLAS patients. Comparison of non-renal manifestations between the two groups shows that XLAS patients in the group promptly diagnosed correctly were more typical, because of more male XLAS patients with sensorineural deafness and more female XLAS patients with typical EM and COL-IF changes (see Table 1).

The misdiagnoses identified in this cohort of patients were often a reflection of histological misclassification based primarily on light microscopic findings, and failure to perform or accurately interpret EM and collagen IV studies [18,21]. Light microscopy is widely regarded as of limited value for differentiating between primary glomerulonephritis and AS [22,23]. Diffuse mesangial proliferative changes were observed in most AS patients (ARAS patients and all male XLAS and young female XLAS patients). Segmental changes (either segmental proliferation or focal global/segmental glomerulosclerosis) were more frequently found in advanced AS patients and female XLAS patients [24,25].

We did not find significant differences between the two groups with respect to the presence of non-specific diagnostic lesion, including basement membrane thickness in GBM or Bowman’s capsule, foam cell clustering in the interstitium, and pauci-immune feature in routine immunoflurence studies. Non-specific immune deposits were observed in a few patients, but more specific patterns of deposition were observed only in those with coexisting renal conditions, such as primary IgA nephropathy, idiopathic membranous nephropathy, lupus nephritis, diabetes, primary hypertension, hepatitis B virus infection, pregnancy-related nephropathy, and drug-induced tubulointerstitial nephritis. More difficulties in diagnosis were found in female XLAS patients, with frequent misclassification as focal segmental glomerulosclerosis, mesangial proliferative glomerulonephritis, minimal change disease, and thin basement membrane nephropathy.

The procedures used to correct the diagnosis in misdiagnosed patients are described in Table 2 with one primary procedure, with or without secondary procedures. Re-biopsy with complete evaluation of EM and COL-IF was the most frequently used procedure for correcting the diagnosis in male XLAS patients and ARAS patients whose family history was atypical or difficult to obtain, and whose first biopsy samples were not available for EM and COL-IF studies.

Most re-biopsied patients were referred patients without data of EM and COL-IF studies in their initial biopsy performed in local hospitals. Re-biopsy usually showed typical changes in both EM and COL-IF studies, as well as more prominent non-specific lesion (including thickened GBM and laminated Bowman’s capsule and more severe clustering of interstitial foam cells). Skin biopsy for collagen IVα5-chain immunofluorescence study is complementary in diagnosing XLAS, although it may be less sensitive in female patients [26,27]. Early stage AS patients may present as thin basement membrane disease, with or without messangial lesion. This is especially common in female XLAS patients, in ARAS patients, or AS patients with compounded mutations in COL4A3 and COL4A4. Progression of renal lesion from thin basement membrane disease to focal and segmental glomerular sclerosis has been observed and reported in both XLAS patients and ARAS patients diagnosed with genotyping [24].

Diagnostic criteria for AS were first presented by Flinter and colleagues in 1988 and were widely accepted during the years followed for a clinico-pathologic diagnosis [15]. Efforts have been made to include genotyping and COL-IF study as more specific diagnostic criteria, and more patients from AS families are thus diagnosed without resort to renal biopsy. Over the last 20 years when renal biopsy played an essential role in the initial evaluation of AS patients, genotyping has been accepted as a gold standard in AS diagnosis and family counseling worldwide, with the growing availability of the technique to clinic. Typical EM changes in GBM are considered as pathologically specific in patients evaluated with renal biopsy, and COL-IF studies (of renal and/or skin samples) are accepted as molecularly accurate diagnostic criteria besides genotyping for AS diagnosis [28-31].

Family history is also an important clue leading to direct work-up with planned order for COL-IF testing at initial evaluation. However, in our study, difficulties existed in collecting family history data in both the misdiagnosed and the promptly diagnosed groups. There were 13 patients (25%) in the misdiagnosed group and 15 patients (28.8%) in the promptly diagnosed group whose family history was negative or atypical. Family history may be absent in patients with de-novo mutations, or atypical in patients from small size families, or may be difficult to obtain for cultural and social reasons. There were 26 patients in the previously misdiagnosed group whose family histories were discovered only during the second evaluation with renal biopsy or during long term follow-up after the first evaluation. In 8 female XLAS patients, positive family history was available only after a family member was later diagnosed with pathologic evidence of AS, which was important for correction of previous misdiagnosis in these patients [32].

Difficulties also existed in evaluating both the EM and the COL-IF studies. Thick and splitting GBM under EM is the most typical diagnostic feature before genotyping is conducted, but there were several cases in which changes of thin basement membrane nephropathy and membranous nephropathy were difficult to differentiate [19]. Attenuated staining with monoclonal antibodies for the sub-chains of collagen IV has been found in some patients who manifested atypical in COL-IF studies. In clinical practice, delayed reports, inadequate biopsy sampling, technique failure, and unavailable facilities for complete studies in some hospitals frequently contributed to misdiagnosis. In female XLAS patients and younger patients, the EM and COL-IF changes might be even more atypical and easily overlooked [33].

Although changes by light microscopy alone are not specific for the diagnosis of AS, important pathologic clues included pauci-immune deposits associated with minor glomerular change, thick or laminated walls of capillary and Bowman’s capsule, clustered foam cells in interstitial area that should remind pathologists and nephrologists of the possibility of the syndrome and the need for timely EM and COL-IF studies plus clinical evaluation of ears and eyes [16,34-36]. Non-specific clinical clues such as progressive proteinuria and sustained hypoalbuminemia were frequently observed in long-term follow-up and also used as clues to correct misdiagnosis [37,38].

In practice, there is usually a period of days or weeks for collecting diagnostic data for AS. Even after evaluation of the renal biopsy in some patients, there is a “mid-diagnosis” period for further investigation of family history, for waiting the EM report, for further study of collagen IV chains in renal biopsy or further ordering of skin biopsy, and/or for a genotyping study of the family. In the misdiagnosed group, patients previously misdiagnosed as thin basement membrane nephropathy had this period of mid-diagnosis.

Including COL-IF as an additional diagnostic criterion, we analyzed the number of reached criteria and the odds ratio of misdiagnosis. We found that AS patients with fewer than three diagnostic criteria had a 3.29-fold risk for misdiagnosis, and the OR reached 3.90 in female XLAS patients (Table 4).

In China, more and more hospitals and institutions are able to perform renal biopsy and routine pathologic evaluation. However, there remains a great need for cooperation and development, including a better referral system to share pathologic and genetic information [37-39]. The CASTOR e-system was developed for this purpose and to facilitate better care of AS patients in China. Collaboration through an effective e-system will not only reduce the chance of misdiagnosis but also provide a good research platform for AS treatment studies [40-43].

This study has several limitations. First, it did not cover young children, and the patients involved were of adults and adolescents, thus increasing the proportion of patients classified as late onset subtypes. Second, it did not directly analyze the factors leading to misdiagnosis because these direct data of the referred patients were often not available. Third, most of the patients were not diagnosed with genotyping. The information provided in this study indicates that (a) to minimize the chance of misdiagnosis in AS, patient’s data on the five specific parameters listed above must be obtained initially, and (b) atypical cases (especially female XLAS patients) with less than 3 of these diagnostic criteria are at greater risk for misdiagnosis.

## Competing interests

All the authors declared no competing interests.

## Authors’ contribution

All the authors contributed to the clinical and pathologic studies of the cases involved. XDY designed the study. ZHL supervised the study design. XDY, XC, GYH, YTY, STX contributed to data collection and Statistical analysis. XDY drafted the manuscript. All authors read and approved the final manuscript.
